# How can community music shape individual and collective well‐being? A case study of a place‐based initiative

**DOI:** 10.1002/hpja.921

**Published:** 2024-09-10

**Authors:** Emma Heard, Brydie‐Leigh Bartleet

**Affiliations:** ^1^ Creative Arts Research Institute Griffith University Meanjin/Brisbane Queensland Australia

**Keywords:** community, health equity, health promotion, inequality, music, social determinants of health, social equity

## Abstract

**Issue Addressed:**

There is an urgent need to investigate innovative and creative approaches in health promotion that support work towards health equity. In response, this study explores the potential for arts, and community music specifically, to strengthen individual and collective well‐being.

**Methods:**

This study used a qualitative case study methodology that involved interviews, focus groups and ethnographic observation with participants (*N* = 13), facilitator (*N* = 1) and support staff (*N* = 2) of an established community music initiative conducted in an urban community. Data collection was conducted across 2023 and data analysis drew on a socioecological framework to explore potential individual and collective outcomes from the perspective of those involved in the initiative.

**Results:**

Findings point to outcomes across socioecological levels with researchers identifying positive health and well‐being implications for participants including joy, healing and a sense of purpose, creative self‐expression, confidence, social connection and contribution. Positive outcomes for the wider community were also identified including developing community ties, promoting safety and shaping and sharing of collective identity. Findings suggest community‐led opportunities for engagement can support healing and empowerment for people who are marginalised, and this can enable active community participation related to challenging the status quo and developing a shared set of values. Potential implications of these outcomes in relation to broader societal transformations are discussed.

**Conclusions:**

This study highlights how community music, and the arts more broadly are working in communities in ways that support potential personal, community and societal transformations towards health equity.

**So what?:**

By developing coalitions and collaborating with diverse sectors, including the arts and social sectors, health promotion practitioners and researchers can harness the creative strengths and resources that exist within a community to support positive individual and collective well‐being.

## 1 BACKGROUND

Health inequity is a growing challenge globally, including in Australia, and there are calls for greater attention and action towards addressing the social determinants of health, that is, the contexts within which people are born, grow, live, work, play and age.^1^, ^2^ The social determinants of health can account for more than 50% of health outcomes, and as a result non‐health sectors have a significant impact on population health.^3^ This demonstrates the need to expand traditional conceptualisations of ‘health’ and ‘health care’ to embrace socioecological models that see people and their health practices as situated within and shaped by broader social, economic, political and cultural contexts.^4^, ^5^, ^6^ Since its inception, the field of health promotion has been grounded in a socioecological understanding that sees action across individual, community and society levels as essential for promoting health and well‐being for all.^7^ Well‐being is ‘a positive state experienced by individuals and societies.’^8^ ^(p10)^ The World Health Organization recognises well‐being as a resource for daily life and highlights how a focus on well‐being is important for understanding and addressing health equity.^8^

The arts are emerging as powerful approaches for health promotion action that can support transformative change across individual through to societal levels.^9^, ^10^, ^11^ Within the broader field of the arts, there is compelling evidence to suggest music can help in achieving therapeutic, public health and health promotion outcomes.^10^, ^12^, ^13^, ^14^, ^15^, ^16^, ^17^ Currently, music, health and well‐being initiatives are occurring across a vast number of contexts, including therapeutic, community, education, clinical and everyday settings from schools to palliative care wards, prisons and public spaces.^18^, ^19^ Together, these highly diverse approaches to using music provide a compelling picture of how music can be widely applied to achieve outcomes that bridge many interconnected facets of health and well‐being.^15^

In general, research in this broad field has focused most strongly on singing,^14^ which offers a wide range of benefits including relaxation, emotional release, stress reduction, happiness, stimulation of cognitive capacities, connection with others, sense of contribution, confidence and self‐esteem, as well as perceived physical benefits.^20^ A wide range of outcomes have also been studied across instrumental and music‐making contexts, styles, traditions and cultures.

Building on a long history of practice and research within the field of music therapy, recent decades have seen a proliferation of practice beyond clinical settings to include community music activities that seek to contribute to broader individual and community well‐being outcomes. Within this broader landscape of music and well‐being practices, sits the field of community music. Community music can be broadly defined as participatory music making by, for and/or with a community. At its heart, community music involves the creation of inclusive, locally embedded, community‐led opportunities for engagement in music.^21^ These outcomes range from individual (e.g., relating to identity, physical coordination, emotional regulation and the cognitive) to collective (e.g., nurturing relationships with others, fostering of social connection and a sense of community).^18^ However, there are still gaps in our understandings about how positive outcomes at the individual level can flow upstream to the kinds of macro, systemic changes needed for health equity to occur.

To address this gap, Bartleet (2023)^22^ draws on a socioecological understanding to create a framework for exploring how community music might promote four dimensions of outcomes: individual, micro, meso and macro. In Bartleet's (2023) framework, the individual level refers to *personal transformations* including aesthetic pleasure and creative expression, cultural connection and emotional well‐being, identity affirmation and confidence, as well as personal safety and self‐awareness. At the micro level, community music can support *relational transformations* including social connection and friendships, social integration, recognition and participation and dialogic interactions. Building on these individual and interpersonal outcomes, community music can also lead to *community transformations* at the meso level by supporting collective identity and shared culture, sense of belonging, social capital (bonding, bridging and linking) and shared understandings. Finally, Bartleet (2023)^22^ suggests community music may also be influential at the macro level supporting *structural transformations* related to ideologies and discrimination, social, economic and political orders, human rights, public policies and safety.

Informed by Bartleet's (2023) Dimensions of Social Outcomes in Community Music framework,^22^ this study aimed to explore the potential of an established, long‐term community music initiative to affect health and well‐being through personal, relational, community and structural transformations. From a place‐based perspective and focusing on participants' experiences and perceptions, we sought to identify potential well‐being related outcomes, as well as challenges, emerging from the initiative.

### 1.1 The Whoopee‐Do Crew case study

This study investigates the impact of an established, long‐term community music initiative, the Whoopee‐Do Crew. The Whoopee‐Do Crew has been running for more than 10 years in Kurilpa, Meanjin (Brisbane, Queensland). Kurilpa is an urban, inner‐city suburb facing a unique set of social and economic circumstances including rapid gentrification, climate‐related disasters (such as flooding and heat waves) and a growing cost of living crisis putting families and people at risk of and into homelessness.^23^, ^24^, ^25^, ^26^ Additionally, this inner‐city community will be a central location for the 2032 Olympic Games, which is exacerbating tensions and pressures related to housing costs, social services and amenities and (over) development in the area.^27^ Founded by musician Tom Smith, the Whoopee‐Do Crew is a community‐led initiative currently facilitated by a well‐respected First Nations musician and community development worker, Jenny ‘Pineapple’ Martinelli. The group meets weekly in a central public space, locally known as ‘People's Park’ or ‘Little Park’ on Boundary Street—the main independent commercial strip in Kurilpa. Boundary Street is significant as it historically marked the racist segregation perimeter where First Nations people were excluded from.^28^ Kurilpa remains a culturally significant area for First Nations Peoples, including Turrbal and Yuggera/Jagera/Yaggera Peoples, with the local Musgrave Park being a historical meeting place and contemporary site of protest, celebration and political movements. People's Park is an important community space and regular meeting place for the First Nations community.^29^

The Whoopee‐Do Crew is an inclusive healing space that welcomes all members of the community to make music together. Sessions are opened and closed with a song in Yuggera language and everyone present contributes to the session by playing instruments, singing, setting up and or packing down or simply offering a ‘sound’ at the opening of the session. The group is transient and eclectic, made up of professional and semi‐professional musicians, community members with an interest in music or wanting to learn an instrument, people experiencing homelessness and/or poverty, people with disabilities and their support workers, people with mental health challenges, people experiencing or at risk of social isolation and loneliness and people experiencing other forms of marginalisation. Consistent with international community music practice^21^ and intersectionality informed health promotion,^6^ the sessions are conducted with a commitment to hospitality, inclusion and recognition of the capabilities and strengths people bring from their diverse life experiences and social positions. Everyone is welcome, including passers‐by who might stop to listen for a moment or join the sessions for any amount of time, with no rules or limitations beyond safety and respect for self and others (including no smoking or drinking alcohol in the circle). The group maintains a non‐hierarchical ethos with decision‐making and contribution shared among all present. When the facilitator is absent, the group continues to run with different members stepping up to lead the welcome and closing, and the group deciding together which songs to play.

The group plays mostly original songs that have been collaboratively written over many years, the majority from the founding days when Tom Smith worked closely with members of the community to turn their stories into songs. While the group does not actively seek opportunities to perform publicly, they are regularly invited to perform at a range of community events including social sector events, community celebrations and days of national significance, for example, during NAIDOC—Australia's annual program to celebrate and recognise the history, culture and achievements of Aboriginal and Torres Strait Islander peoples. All members of the group are invited to be a part of the performances and are encouraged and supported to sing and play regardless of their ability. The Whoopee‐Do Crew is unfunded but connected to the local neighbourhood centre, Community Plus+ West End Community House, which provides support through a First Nations community development worker who offers assistance with logistics and is present during the sessions to support the facilitator and offer individual assistance and referrals to members of the group where necessary. In Australia, neighbourhood centres are commonly funded by State Governments. They are responsive to the unique community needs in the particular area they service, but generally provide social infrastructure, support community connection and cohesion and facilitate services and support for people experiencing a range of hardships.

## 2 METHODS

This study was conducted within the Creative Change Project (www.creativechange.org.au), an Australian Research Council funded project. The project received ethical clearance from Griffith University's Human Research Ethics Committee (2020/679). The lead author is a qualitative health researcher who has lived and worked in Kurilpa most of their life; the second author is a leading community music scholar who has a migrant background but has worked in Kurilpa for 19 years. Both authors' work is shaped by social justice and a constructivist approach that frames knowledge production as socially constructed.

This discrete study used a qualitative case study methodology to explore how people involved in the Whoopee‐Do Crew perceive potential outcomes related to individual and collective well‐being. Semi‐structured interviews with the group facilitator (*N* = 1) and neighbourhood centre staff (*N* = 2) were conducted to understand their perspectives about the outcomes of the group for people participating and the value of the group in relation to the broader community. The facilitator, who had initially been a participant, stepped into the facilitator role volunteering their time for the past 6 years. They are an established musician in their own right, have a life‐long history of community development and social work and they are deeply engaged with the Kurilpa community. The two neighbourhood centre staff were both involved in supporting the Whoopee‐Do Crew activities at different times and know many of the participants from their engagement in other activities connected with the neighbourhood centre. Across 2023, the researcher for consistency participated in weekly Whoopee‐Do Crew sessions and conducted semi‐structured interviews, focus groups and ethnographic observation. Six people identifying as women and seven people identifying as men, who had been involved in the Whoopee‐Do Crew for between 1 and 10 years, participated in interviews and focus groups. These were conducted in an informal manner, before, during and after sessions with questions designed to explore people's experiences of participating in the Whoopee‐Do Crew and understand their perceptions about the potential benefits, challenges and outcomes of the group for themselves and the broader community. Questions included ‘Why do you think this initiative is important’, ‘What do you and others get out of this initiative?’, ‘Does this initiative play a role in the West End community, how/why?’ To accompany this data the Research Fellow wrote field notes after Whoopee‐Do Crew sessions that described key moments, interactions and conversations. All interviews and focus groups were transcribed verbatim and field notes that were also included as data.

### 2.1 Data analysis

We took a thematic approach to data analysis, which aimed to explore the subjective experiences of people involved in the Whoopee‐Do Crew. Coding involved a three‐step, layered process^30^ that began with Emma Heard familiarising themselves with the data through an in‐depth reading of each transcript and field note entry making notes related to key ideas and points of interest in the data. Emma Heard then conducted an initial round of open‐coding, inductively identifying experiences, outcomes and challenges of participation described in the data. These initial codes were shared with the Brydie‐Leigh Bartleet, who offered validation and insights from their experience as a musician and established scholar in community music and social impact. The third step in our coding process involved revisiting the coded data, looking for similarities and connections between codes in order to categorise and combine individual codes. Emma Heard and Brydie‐Leigh Bartleet worked together to generate themes from these categories, informed by Emma Heard's health promotion background, Brydie‐Leigh Bartleet's community music background and both authors' social justice framing for understanding social change. In order to contribute to both health promotion and community arts disciplines, our final thematic organisation was guided by emerging literature in these fields,^9^, ^11^, ^18^, ^31^ specifically, the Dimensions of the Social Outcomes of Community Music framework.^22^ Drawing on an ecological understanding, this framework allowed for exploring perceived outcomes across individual, relational, community and societal levels of change in related and nested ways. The final thematic analysis was shared with the facilitator and neighbourhood centre staff, who provided insights and corroboration from their experiences in social service provision, community development and community arts facilitation.

## 3 RESULTS

We identified themes relating to individual, relational, community and structural transformations as outlined in Bartleet's (2023) Dimensions of Social Outcomes in Community Music framework.^22^

### 3.1 Individual transformations

Data demonstrated how people involved in the Whoopee‐Do Crew perceive the initiative to support a range of positive outcomes for individuals related to personal well‐being including joy, healing, physical health, a sense of purpose and a way to contribute. Creative self‐expression appeared to support these well‐being outcomes as well as a sense of belonging. Additionally, data suggested the building of confidence through mastery and skill development which extended into people's lives beyond participation in the Whoopee‐Do Crew.

#### 3.1.1 Well‐being

**FIGURE 1:**
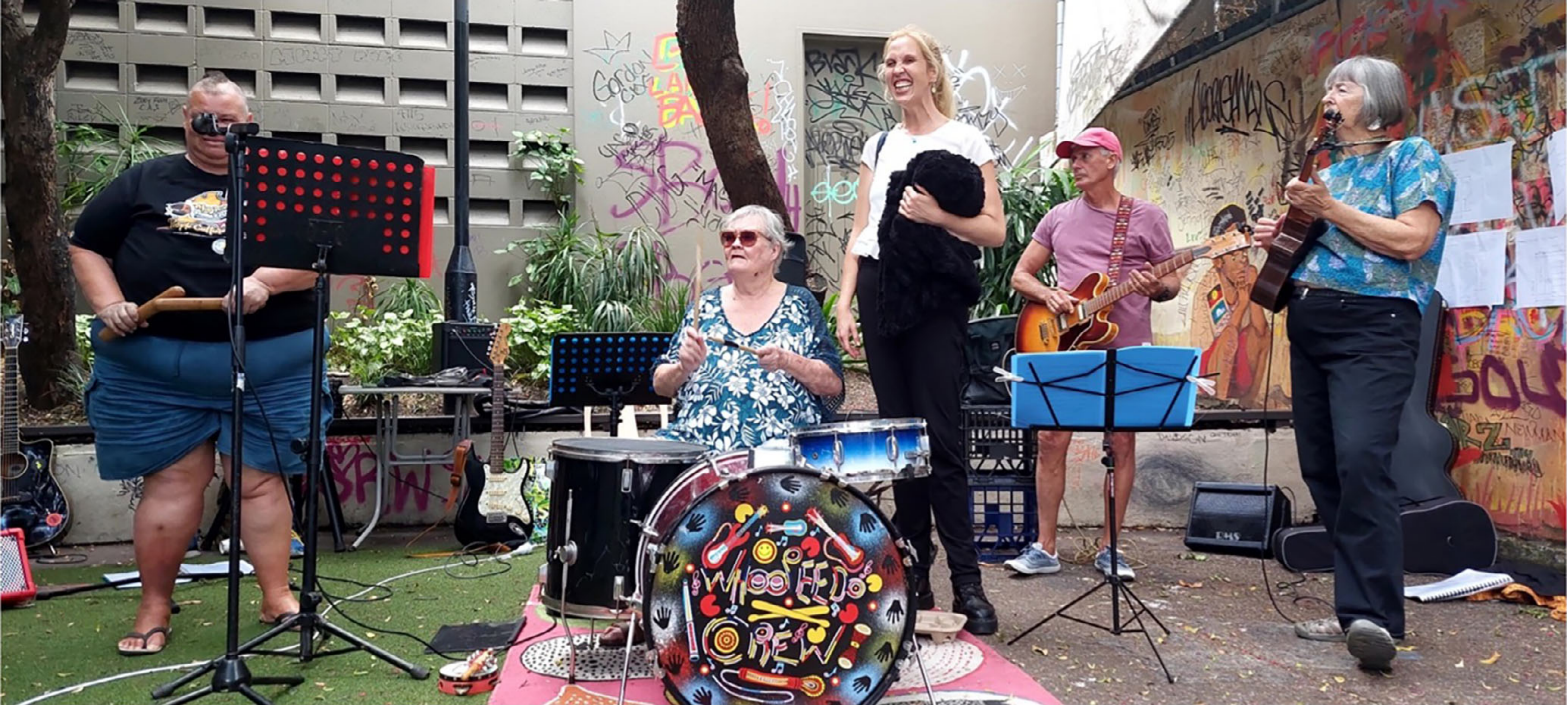
Demonstrating joy at a Whoopee‐Do Crew rehearsal (photo by Emma Heard).

Observational data demonstrated that during any Whoopee‐Do Crew session, the joy created among the group was palpable, with smiles, laughter and light‐hearted banter (demonstrated by Figure 1). Almost all Whoopee‐Do Crew participants mentioned how happiness was an important part of their experience and for some participants, this opportunity to participate in something joyful with others was rare and valued.

It just gets me out of the house for a couple of hours, gives me something to do, it's something I enjoy doing, muck[ing] around with music. (Whoopee‐Do Crew participant, man, W311)

Wednesdays are a good morning! (Whoopee‐Do Crew participant, man, W315)

The researcher observed the way many different people involved in the Whoopee‐Do Crew sessions engaged in physical movement (e.g., dancing and percussion) and vocalisation (e.g., singing), including those with a disability. Consistent with Dimensions of Social Outcomes in Community Music, this active participation can be important for supporting physical and psychological well‐being.^22^, ^32^

The facilitator described the Whoopee‐Do Crew as a ‘safe space’, a space for ‘healing’ and ‘recovery’. Three participants discussed how participation supported personal healing and coping with challenging emotions. As one participant stated, ‘It's a safe place to grieve. It's a safe place to … release certain energies [and] to be in [our] bodies’ (Whoopee‐Do Crew participant man, W315).

**FIGURE 2:**
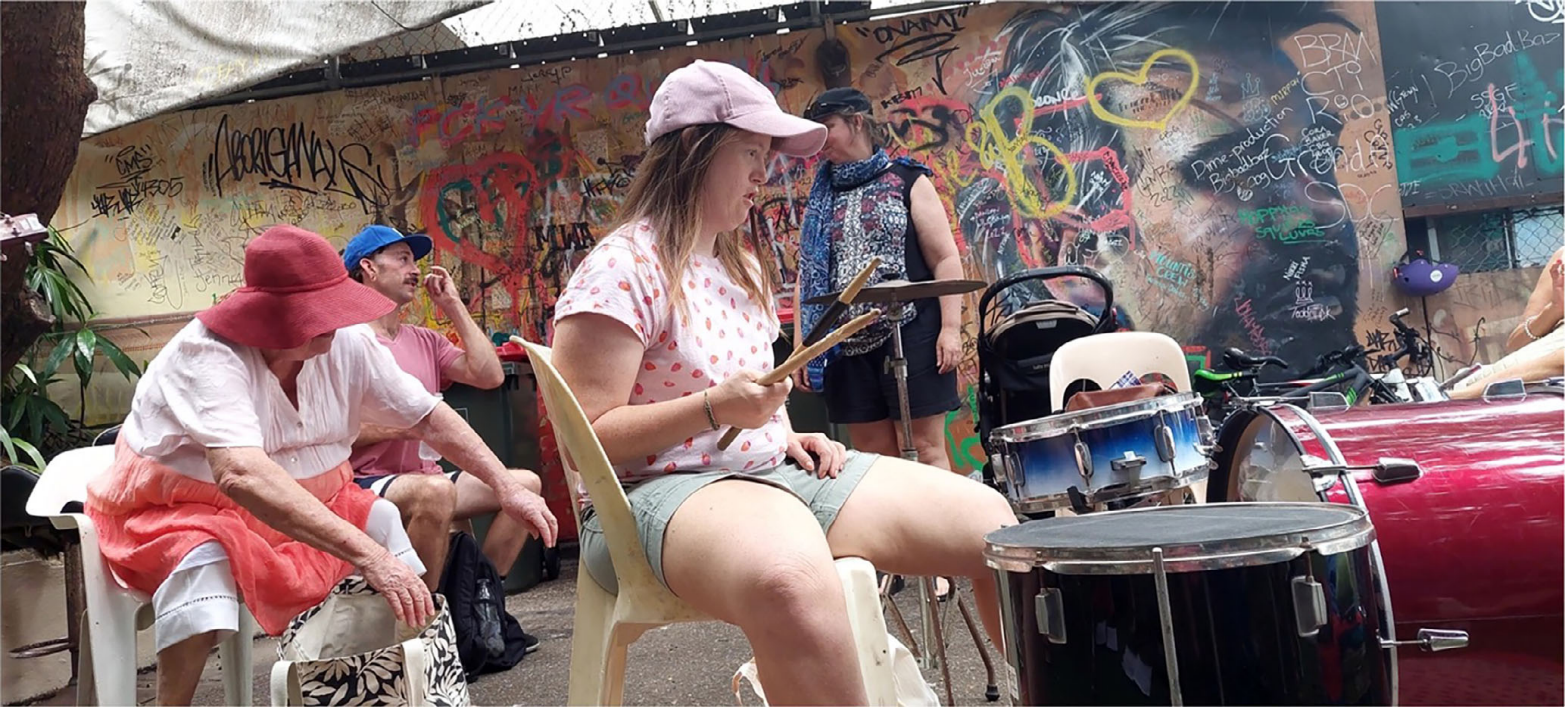
Regular member trying a new instrument and gaining new skills (Photo by Emma Heard).

Some participants highlighted ways the Whoopee‐Do Crew gave them a sense of purpose as well as creating pathways for new opportunities and experiences through skills and relationships developed during the sessions. This included being encouraged and supported to learn new musical instruments (as demonstrated in Figure 2), collaborating on new songs and opportunities to perform including at paid performances. Such experiences were described by many different people involved in the sessions including participants, facilitator, neighbourhood centre staff, support workers and the researcher.

I've never sung with a microphone before in my life. I've gone to university to learn to play music … [but] there's a lot of things that I didn't learn at some places that are doing the educational things, that I've been able to learn them at The Whoopee‐Do Crew. (Whoopee‐Do Crew participant, man, W315)

I've been with Whoopee‐Do music crew for maybe eight years and I even just contributed a new song this morning and the band is so good that they can just pick up the chords, I can say give me A‐E‐E‐D‐A and they're on board. That's Whoopee‐Do. They're brilliant. (Whoopee‐Do Crew participant, woman, W316)

#### 3.1.2 Creative self‐expression

Many participants and the facilitator described the Whoopee‐Do Crew as an instrument for making community voices heard—all members of the community, and importantly those commonly silenced. As one participant stated:

So, the gift of Whoopee‐Do Music Crew is the fact that it gives the voiceless and the underdogs a voice and a position to be heard and acknowledged in community. (Whoopee‐Do Crew participant, woman, W316)

**FIGURE 3:**
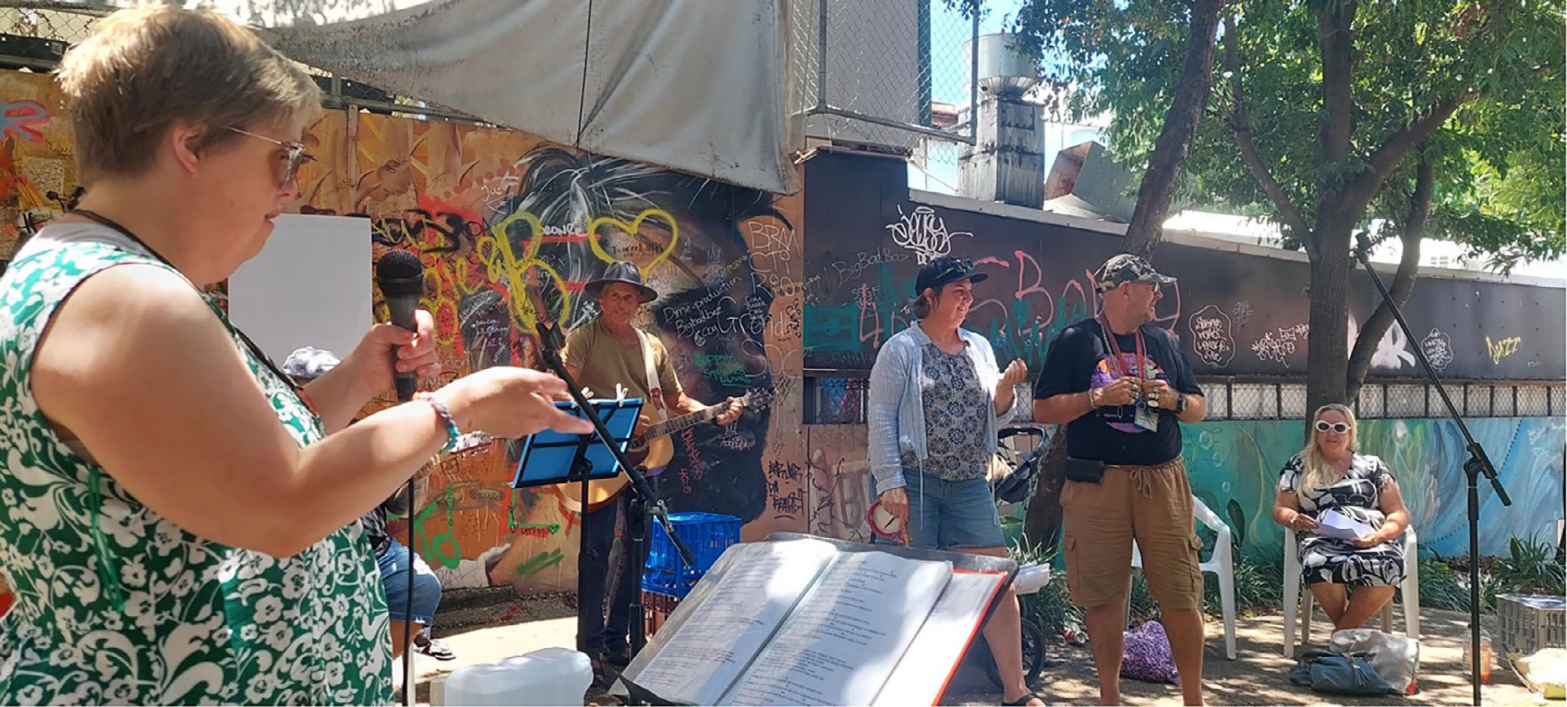
Rare and meaningful moments of self‐expression through song with others (Photo by Emma Heard).

The creative self‐expression generated through collaborative songwriting, storytelling and music‐making appeared significant for many people involved and a key mechanism through which other individual transformations were realised (see Figure 3). As the facilitator explained:

Through self‐expression, that's when you find your voice and feel you're being listened to. You start to heal when you feel you belong. A lot of trauma and a lot of angst and mental health issues, [are related to] people not feeling they belong. … So then you've got to create belonging. So we create belonging through community. (Facilitator, W007)

The facilitator and neighbourhood centre support staff described how they understood participants performing their songs as a way for people involved in the Whoopee‐Do Crew to share their identities, develop strong connections with one another, gain recognition from their community and also educate the community about key issues affecting their lives (such as discrimination). The Dimensions of Social Outcomes in Community Music^22^ describes how levels of transformation are interrelated, and these findings have implications for the community and societal transformations discussed in the following sections. In the following quote, a First Nations participant outlines how songwriting and performing with the Whoopee‐Do Crew is, for them, a form of expression, activism and celebration of people and place.

It's a peaceful way of education. It's a happy way of educating people. And not all Whoopee‐Do Crew participants write down sad songs, there's happy songs. You got [participant's] song, it's a happy song. … Them songs need to be applauded. (Whoopee‐Do Crew participant, male, W115)

Observational data demonstrated that opportunities for creative self‐expression are extended to everyone involved, people are encouraged to bring songs they have written, and the group embark on collaborative songwriting together. For example, during one session that the researcher was participating in, the group composed an instrumental piece for one participant who had challenges with speech, which the group played while the participant improvised spoken‐word poetry over the top. This piece became a regular part of the Whoopee‐Do Crew sessions and the researcher witnessed the participant excited to perform it each week with growing confidence and increased language and flow. The facilitator described opportunities for creative self‐expression as an essential element of the Whoopee‐Do Crew. For the facilitator (W007), “it's [about] dignity … giving [people] the tools to find their own dignity, to find their own voice, find their own expression”.

#### 3.1.3 Confidence and empowerment

The facilitator discussed the importance of their collaborative and non‐hierarchical approach for supporting skill development and confidence to try new things. The researcher witnessed this during a collective songwriting activity where all members of the group were encouraged to bring new ideas for songs and poetry to the group. The facilitator reflected about how the group demonstrated growing skills and enthusiasm for songwriting together. These opportunities for people to experience mastery, build confidence and develop new skills, support empowerment in their daily lives.

So [Participant A who has disabilities] was able to perform [his song] himself. And shine. And having those accolades, he just loved it. … It gives him that boost, so that he goes confidently into society. … I think [performing] gives people the confidence, … gives them more confidence to be in life, and to go round in life and be who they are. Because when they're accepted on stage, then it's like, well, ‘you can accept me all the time then’. (Facilitator, W007)

Almost all participants discussed how they had gained confidence from being a part of the Whoopee‐Do Crew and many highlighted how they value the sense of achievement and recognition from their community that comes from opportunities to perform publicly with the Whoopee‐Do Crew.

Performing, you feel that you earn kudos for being here. Now this is a public space, so I get people coming up to me now and saying, ‘Wow, you really rock on that [instrument]’ and that is really important to me. (Whoopee‐Do Crew participant, woman, W310)

While publicly performing appeared to play an important role in outcomes related to confidence and empowerment, the facilitator discussed practical and logistical challenges in facilitating performances with such a diverse group, many of whom require support to travel to and participate in sessions and performances. These multifaceted benefits for individual people involved are demonstrated by the visible retention within the group. Yet, while the open nature of the group was a clear strength in terms of inclusion and accessibility, the transient nature could also be a challenge for collaborative songwriting and performances.

People go ‘Oh, I'm gonna come back’ and then some come back for a while, then they go again, the challenge is that it's transient. (W007, Facilitator)

### 3.2 Relational transformations

Data suggested that through engagement with the Whoopee‐Do Crew, people from all walks of life, backgrounds and life situations develop strong bonds that lead to emotional and practical support. Authentic inclusion fostered by the facilitator is integral for developing and maintaining this connection.

#### 3.2.1 Inclusion

**FIGURE 4:**
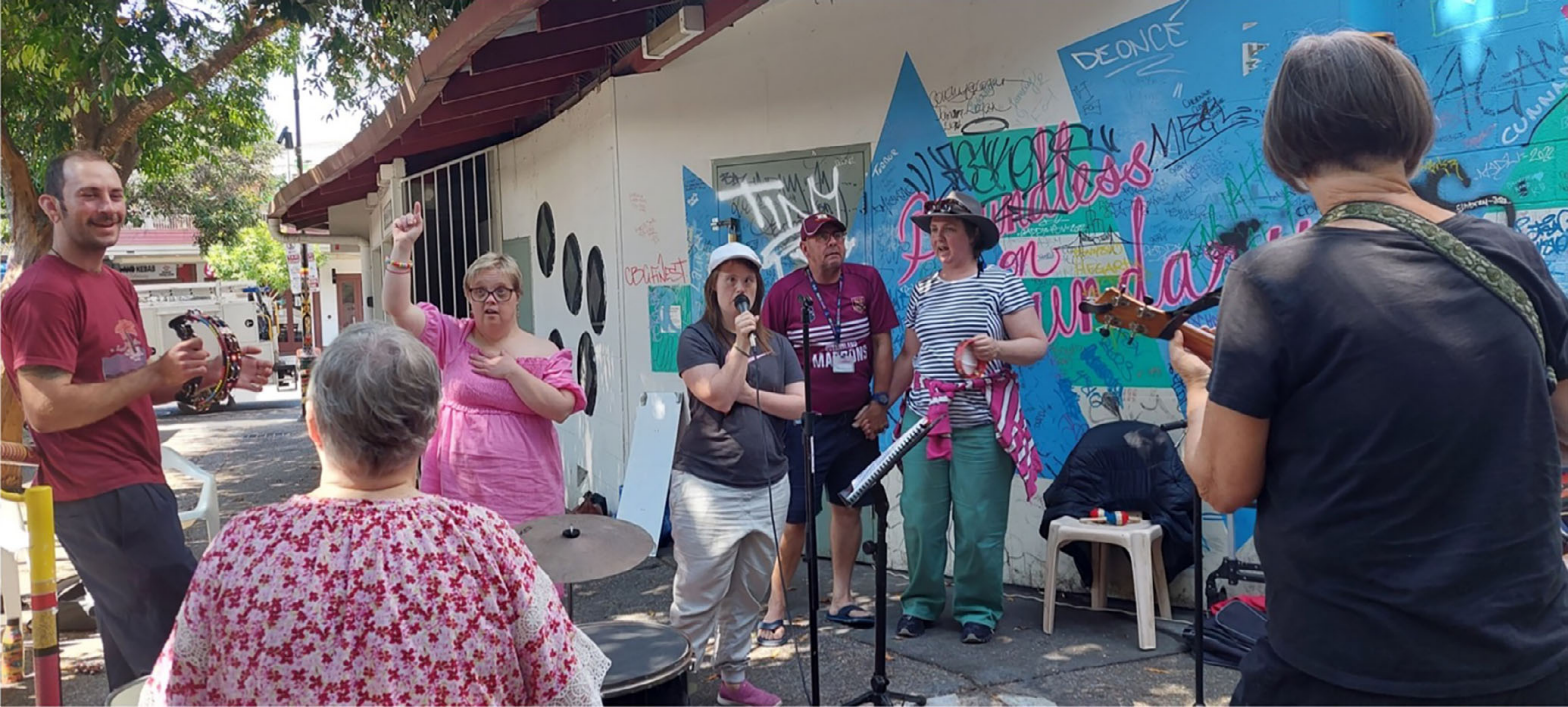
Inclusive spaces and strong bonds created at the Whoopee‐Do Crew rehearsals (Photo by Emma Heard).

The Whoopee‐Do Crew is an open space for anyone to join and be involved. People involved are from a wide range of backgrounds including professional and semi‐professional musicians, people with disabilities, people experiencing social isolation and loneliness, people wanting to learn an instrument, and people living with homelessness and other social and personal challenges. Participants described how they enjoyed music and observational data demonstrated how everyone who participated in the sessions were encouraged and supported to sing, play, dance and at a minimum contribute to the opening of the session with a ‘sound’ (as demonstrated in Figure 4). This authentic inclusion^21^ is an integral aspect of the group as it facilitates participation based on mutual respect and enjoyment, and data from a range of participants suggested this is something some members do not get to experience often in daily life.

There's no inequality here. Most things [participant] goes to are for people with a disability, but The Whoopee‐Do Crew is for everyone. (Whoopee‐Do Crew participant, carer, woman, W622)

This sense of inclusion, supported by the facilitator and extended by every person involved, is demonstrated in the following comment by a person new to the group, someone who was experiencing a range of physical, social and emotional challenges, “I'd heard about this but was a bit shy to come along. I really appreciate how welcoming you all are, and I can feel the heart” (Whoopee‐Do Crew participant, man, W626).

The facilitator reflected on challenges that people experienced in relation to participation, including mental and physical health and specifically discussed how changes in local social services had affected some people's ability to come to the group and access similar support more broadly.

We have had people drop off. Like when [a particularly local social service organisation] stopped and the [Federally administered National Disability Insurance Scheme changed the way disability support was accessed]. … There [used to be] support workers at [that social service] that supported this sort of thing. (Facilitator, 007)

Being connected with the neighbourhood centre was identified by the facilitator as integral to support participation. The neighbourhood centre staff had established relationships with community members and could encourage people interested in music to attend Whoopee‐Do Crew sessions and provide care and referrals to anyone who needed support before, during and after the sessions.

[The neighbourhood centre provide] tea and coffee, but you know, like, someone [can ask staff when they] can't get on to Centrelink [Federal welfare system] or whatever. [Staff] might bring people up from [the neighbourhood centre] or send people up to the centre from here. So be coordinated with the centre [is really important]. (Facilitator, 007)

#### 3.2.2 Contribution

Contribution was highlighted as an important aspect of inclusion at the Whoopee‐Do Crew with everyone involved having opportunities to give something, feel valued and have a meaningful role in the group. For example, observational data demonstrated that everyone, including people who have challenges with speech, sung with the microphone with nods of encouragement, expressions of gratitude and cheers extended to all who performed. Others played quietly in the background and their contributions were always acknowledged by the facilitator and other members of the group. For others, their contribution related to setting up and packing down, dancing to music, teaching others or fixing instruments—each of these roles appeared essential to ensure the group works and, for many people, these opportunities appeared to create a sense of purpose, which is an important part of their well‐being. The important role of contribution for well‐being is demonstrated in an offer from a person who joined the group for a few weeks after passing by and hearing the music. This person noticed one of the instruments was broken, fixed it temporarily and said, ‘I can get you a new [instrument]. I might be homeless, but I can do that. I would love to do that for you guys’ (Whoopee‐Do Crew participant, man, W627).

#### 3.2.3 Strong bonds and relationships

Participants described how a regular, ongoing space of inclusion and participation plays an important role in the development of bonds between people involved. As described by one long‐term participant:

Well, especially as I say, with people who have got, you know, limited socialisation… I think it helps a lot with bonding. Especially the socialisation bit because as they come here week by week, and they get to know different ways of doing things. And we sort of, you know, build, we bring [each other] out of [ourselves] (Whoopee‐Do Crew participant, woman, W310)

Through music, people share their stories with each other; stories of trauma and hardship, stories of love, stories of ‘mundane’ aspects of day‐to‐day life, stories of joy and stories of sorrow. And this creates a unique avenue for people to get to know each other with an intimacy that for some participants is seldom experienced in other contexts and relationships. This was described by both neighbourhood centre support staff:

And people's songs and their stories, like some people who haven't been able to communicate, for whatever reason, there's been songs written about their story. Like [participant] has a song about going [to the beach], like people who you see every single day and you don't know these things, [or their] challenges, but when you hear their song, you know a little bit more about them. (Neighbourhood centre staff, W108)

Yes, it's purpose and connection. (Neighbourhood centre staff, W109)

Participant data highlighted how being involved in the Whoopee‐Do Crew creates strong bonds between people, including people from different walks of life who may not otherwise have an opportunity to interact. These bonds have developed into lasting friendships through which people support each other emotionally and practically with life challenges and sharing in life's joys and celebrations.

For me, I've lived in West End since 1987. And I own my own house. In my past I was an academic. I'm actually seeing another side of West End [Kurilpa] that I've never seen before. So, like it's been a bit of eye opener. And making friends with people, I wouldn't get a chance to meet just because we're not in the same circles. (Whoopee‐Do Crew participant, woman, W314)

I think it's just the camaraderie, the closeness, you know what I mean? With all of them. We are not just musicians, we're family. Someone will come in and say, ‘I'm off par today’. And we'll support each other. (Whoopee‐Do Crew participant, woman, W310)

The Whoopee‐Do Crew also appears to create a unique opportunity for support workers and the people they care for to connect and share a mutual interest, participating in something together as equals. The facilitator discussed how participating in music together is an important opportunity for carers and the people they support to develop rapport and mutual respect, which is integral for working together. Further, through the Whoopee‐Do Crew, support workers have developed relationships with each other and are able to support each other.

There's the relationship between support worker and client as well. [Support workers who come have their] own story. So, you know, and I think for us, it's like, including the support workers too. (Facilitator, W007)

### 3.3 Community transformations

Being in a public space, the Whoopee‐Do Crew sessions are a unique opportunity for the broader community to connect and experience joy together. Activating community spaces is key for safety and also has important implications for social cohesion and the development of shared identity. The Dimensions of Social Outcomes in Community Music identifies how such outcomes work to create community transformations that can support more equitable places.^22^

#### 3.3.1 Community connections

At almost all Whoopee‐Do Crew sessions the researcher observed a number of passers‐by stop to listen and leave with a smile. This included people shopping, commuting to work, taking children to school or other outings, council workers, people sleeping rough, local business owners and many others. Participants discussed how the public display of ‘who we are’ played a role in community identity and sense of place.

And I think it kind of makes West End [Kurilpa] more interesting when somebody just walks down the street and they come across a live band, like in the park. … It becomes part of the social fabric or social identity of West End, [including for] people who happen to be visiting. I think it's an important part of that. (Whoopee‐Do Crew participant, woman, W310)

The benefits of these moments of connection are important for social cohesion—rare and unique opportunities for the wider community to see each other and learn about each other. As one participant stated:

“It is good for our community because [we all] understand a lot more about West End [Kurilpa] and people who live here”. (Whoopee‐Do Crew participant, woman, W311)

The facilitator highlighted that being in a public place and supporting an inclusive and open space is important for developing understandings about others and the struggles they might be going through, which can have positive implications for empathy and tolerance.

I think that takes away [judgement]. We see people, their behaviours in the streets, and there [can be] a fear around that. … But, music sometimes, you know, people are doing [disruptive or aggressive] behaviour, but then they come into this space. And they just join in. And so the fear of those people starts to go, you start to find out why they have that behaviour. So it's also creating the community and uniting, and understanding people rather than querying them. (Facilitator, W007)

Being a regular fixture in a public space appears to also support connection for others in the community who may be experiencing social isolation, even if they are not actively participating in the Whoopee‐Do Crew. For example, during one session, the researcher witnessed an elderly community member that did not know anyone in the group come with bags of sandwiches to share. This person told the group that in their culture you give food when someone is sick. This person's family member was in hospital, so they wanted to share this food with others, but was socially isolated and without people to share it with. The sandwiches were received gratefully, and the community member stayed for a couple of songs and a chat creating a moment of social connection and happiness.

“All that connection and ties” (Neighbourhood centre staff, W108) developed through these moments are significant for broader social fabric as they facilitate support in other contexts, laying foundations for connection and assistance among the wider community. As one of the neighbourhood centre staff explained, by stopping to listen to the Whoopee‐Do Crew, people become familiar to each other and then offer practical help if they see the same people in need in other contexts.

Like it's that community stuff, by coming here. So when [Participant] goes down the street, somebody who's seen Whoopee‐Do will be like, ‘Oh, hey, [I know you], how are you?’ Like, it's all of that stuff. The foundations have been laid out for us previously. … That's the benefit to this community. (Neighbourhood centre staff, W108)

#### 3.3.2 Place and safety

The Whoopee‐Do Crew is purposively located in an important community space, with significance for the First Nations community and is also a space where the community has recognised the need for activation to support safety and well‐being.^29^ The Whoopee‐Do Crew sessions transform this public park into an open space that provides a regular time where the park is alcohol and other drug free, at the decision and enforcement of the community. Supporting staff from the neighbourhood centre, the facilitator and a number of participants all expressed that they perceive the opening of this space to the wider community as important for the connection and cohesion discussed above and encouraging people to view and use the space in different ways.

It activates the park in a positive way, which is awesome. … Being in a public space, people will walk by and… it just gets them thinking about this space in the right sort of way. (Neighbourhood centre staff, W109)

Participant and observational data suggested that the Whoopee‐Do Crew sessions also provide an opportunity for positive interactions between different members of the community as well as law enforcement, council workers and business owners. For example, the researcher observed how a local police officer regularly visits and engages with the group, getting to know them and other rough sleepers, and showing a different side to themselves through dancing and chatting. This informal relationship development could potentially be important for mediating and facilitating respectful and supportive interactions in other contexts.^33^

Participants described a sense of connection and belonging to the space, and observational data highlighted how people involved in the Whoopee‐Do Crew work to care for the space including keeping it tidy and contributing to local government consultations about improvements. When a tree that shaded the regular practice area was unexpectantly removed by council, the group held a grieving ceremony and sang together about the importance of the natural environment and the impacts of climate change and overdevelopment in the community.

I think for a lot of people it keeps them grounded … it's surprised me in the last 12 months a lot more people have started to understand what this park is all about. Before, I didn't think we'd be able to have a park at all. … but we've got this one back because off all the stuff together. (Whoopee‐Do Crew participant, man, W314)

#### 3.3.3 Valued by community

Data demonstrating the positive feedback from passers‐by in the form of smiles, clapping and vocal encouragement highlighted how the Whoopee‐Do Crew is valued by the community. Over the course of the study, the researcher observed how it was not unusual for people not yet involved with the group to return with instruments or to listen after previously passing by. The researcher witnessed a bus driver who had come to use the public toilets and was compelled to break into dance as the Whoopee‐Do Crew played a song the bus driver knew and loved. The verbal and physical expressions of gratitude were evident. The Whoopee‐Do Crew participants discussed how they in turn value the chance to give something to their community, strengthening their sense of belonging and purpose.

I've heard a lot of people, make [positive] comments. And to see people who are actually drawn in, they stop and sit and listen, and you think, ‘Wow, I'm making an impact here’. It's always complimentary. There's no negatives when they talk about it. And it makes the band come together. (Whoopee‐Do Crew participant, man, W318)

We do get the people from the street that walk past, but they stop in. You know, they pass by and listen to the music and all you can see is a big smile on their face and their body is moving to the beat. That tells us that we're doing something right. (Whoopee‐Do Crew participant, woman, W316)

**FIGURE 5:**
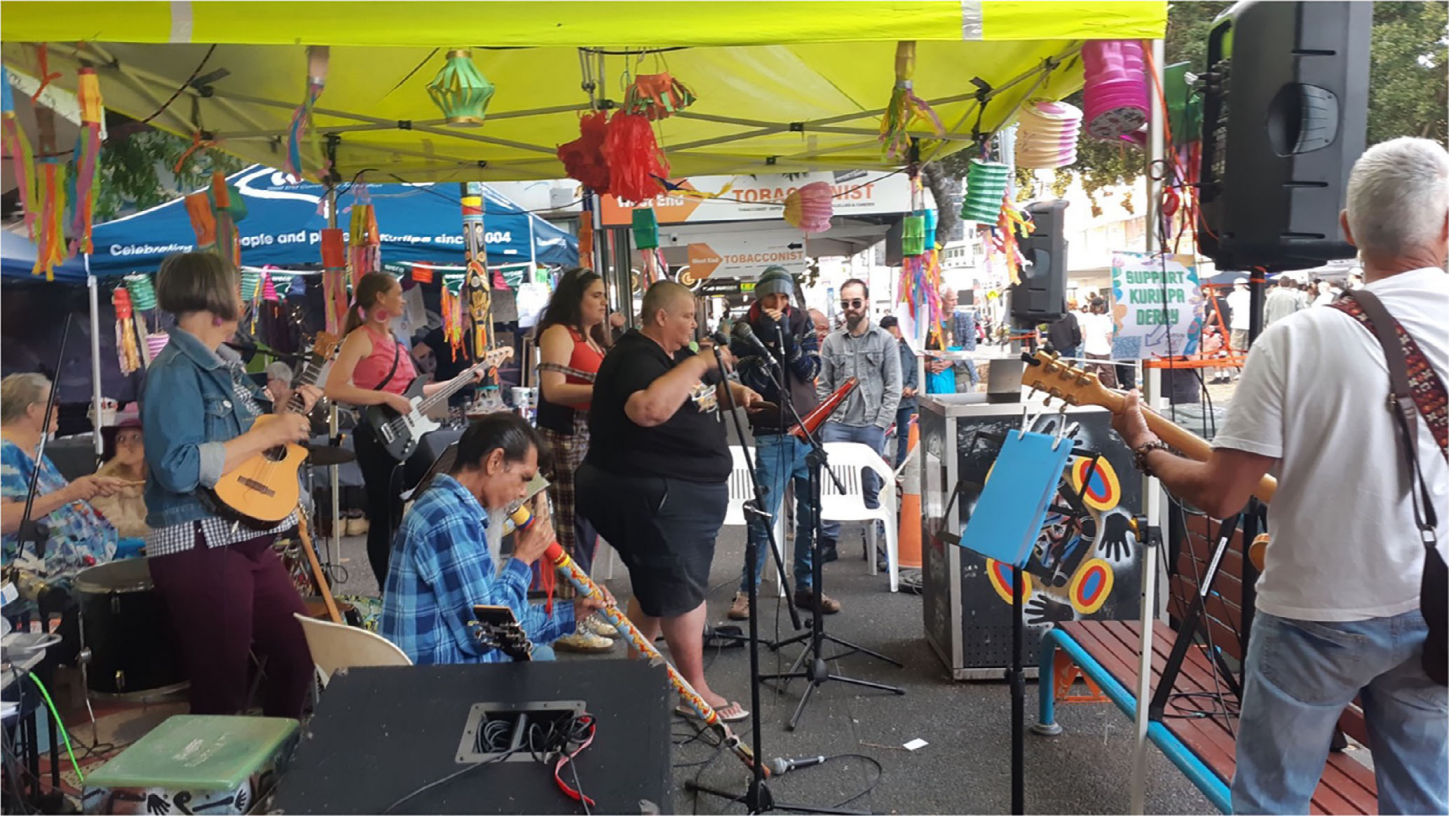
Performance at a community festival (Photo by [Katina Heard]).

The value attributed to the Whoopee‐Do Crew by the wider community is demonstrated by invitations to perform at significant events locally and across Meanjin (Brisbane) (see Figure 5).

We don't actually go out looking for gigs. People hear it and just, you know. Sometimes it's like, musically, not up there, but I think people like the concept of it. I think people, they can feel the energy of it. And they like that. And so people offer us to play. Sometimes paid, some isn't. It's often community stuff. (Facilitator, W107)

A number of participants further described how they see the Whoopee‐Do Crew as contributing to the community's identity. As discussed by a participant below, by being in a public space telling stories, the wider community also gain a sense of belonging and pride from the Whoopee‐Do Crew's sessions.

People know it's happening just by the fact that they are in West End [Kurilpa], and it's like word of mouth or just being aware that it's happening. And you might not even join in but knowing it's happening in your suburb or your community can be uplifting by itself, I think. Even if you don't come up, just by hearing it you think ‘Oh that's happening and that's my community’. (Whoopee‐Do Crew participant, woman, W310)

### 3.4 Societal transformations

Data from this study suggested that the healing that happens through the Whoopee‐Do Crew may also be important for societal transformation. This theory of change was articulated by the facilitator who explained that through opportunities to heal, and become visible and empowered, people who have and are experiencing trauma and oppression gain the capacity to contribute to community, and broader society learns and benefits from these contributions.

Because we've got people healing, then you've got people contributing. And if people are contributing to broader society, then that benefits everyone. … I think it does affect the broader society because when you've got healing happening, that ripple happens. (Facilitator, W007)

Participants also described how the Whoopee‐Do Crew is valued by them as a means through which to demonstrate support for people experiencing hardship, educate broader society about discrimination and other issues, and also to learn about and from the local First Nations community.

So [the Whoopee‐Do Crew are] allowing me to sing educational songs that people don't think they are educational, but they are. It's just that we need to bring those songs up to today. Express those songs. Yes, I'm an activist, but I'm a peaceful sort of activist. … Like I said, it's a peaceful way of education. It's a happy way of educating people. (Whoopee‐Do Crew participant, man, W315)

The importance of such community‐led activism and advocacy that foregrounds the voices of marginalised groups and questions the social power dynamics is commonly unrecognised by public health and health promotion disciplines, but is significant for socio‐political shifts required for equitable societies^6^, ^34^, ^35^ As identified in the Dimensions of Social Outcomes in Community Music^22^ having an awareness of the need to shift ideologies and power structures for greater social equity to occur can be an important precursor for long‐term change in political orders and public policies.

Additionally, for many participants, the Whoopee‐Do Crew is their first and sometimes only exposure to First Nations languages of the area (including the welcome and ending song which is sung in Yuggera with permission from Elders) and being in a culturally significant place facilitates meaningful interactions with, and opportunities to learn from, First Nations Elders and community members. This type of local truth‐telling, sharing and relationship building is central to reconciliation.^36^ Healing and contribution, activism and understanding of historical conditions are theorised to have the potential to contribute to societal change^11^, ^22^, ^36^ and these findings provide insights into how people involved in the Whoopee‐Do Crew perceive the initiative to contribute to societal transformation.

Despite their potential for transformational change at a societal level, the facilitator discussed how community arts initiatives like the Whoopee‐Do Crew have remained on the periphery of much national dialogue about both community development and the arts. This hampers the impact that initiatives like the Whoopee‐Do Crew can have.

Every now and then [community arts] pops up in the mainstream, but then gets buried again, somewhere. And I think that's the thing you know… Sports have always been overriding arts. The arts are [seen as] like a luxury thing. … It's … seen as an elitist thing. And I suppose it is bringing it back to the grass roots [that would support greater impact]. (Facilitator, W007)

## 4 DISCUSSION AND IMPLICATIONS

Drawing on the Dimensions of Social Outcomes of Community Music framework,^22^ this article explores how people involved in one established, place‐based music initiative perceive outcomes suggesting potential to improve individual and collective well‐being. Recent literature has pointed to the under‐acknowledged role of music, particularly for First Nations and Indigenous communities, in attending to the social and cultural determinants of health.^10^, ^16^, ^37^, ^38^ Our findings contribute to this emerging body of literature exploring how community‐centred music making might support well‐being across socioecological levels.

Our findings contribute to an established body of literature demonstrating positive outcomes of collaborative music making in relation to physical and mental health and well‐being, including through expression, connection and confidence.^14^, ^20^, ^39^, ^40^ There is a gap in this evidence base about the benefits for diverse and marginalised groups of people^41^; and, given the backgrounds of participants and complex community context within which this study was conducted, our findings bolster this evidence base. Our findings demonstrate further relational outcomes including developing strong bonds, wider connections and inclusion and contribution to community. This supports an emerging body of literature highlighting the potential for community music to support social capital and contribute to strong community ties, both of which can lead to practical and emotional support as well as civic contribution.^42^, ^43^, ^44^ Providing people experiencing marginalisation and disadvantage with access to these individual outcomes can play a role in mitigating the consequences of health inequity.^31^

At a community level, our findings suggest community‐centred music initiatives can support safety, place identity and contribute to the social fabric of a community. In particular, the open, inclusive design and long‐term, embedded nature of the Whoopee‐Do Crew act as a conduit for people across social divides and diverse backgrounds to become acquainted through a mutually positive experience. Such community connections have important collective well‐being implications and can support community safety.^45^ Further, our findings suggest community music can be a means through which to develop and share a collective identity and emerging literature is demonstrating the importance of addressing place‐based stigma and promoting pride in place for health and social equity.^11^, ^46^, ^47^, ^48^ Finally, our findings point to a potential for initiatives such as the Whoopee‐Do Crew to contribute to societal level transformations, with data suggesting community music may be able to support positive social change through healing and empowerment for people who have been marginalised, providing opportunities to play an active role in contributing to their community and establishing shared values.^49^ In our study, community music was a way for participants to advocate for social justice concerns and engage with First Nations communities and culture. Elements related to healing, storytelling, relationship building and activism identified in this study resonate with calls for better recognition of community‐led advocacy and truth‐telling in efforts towards health equity and just societies.^34^, ^36^ While capturing evidence of societal change was outside the scope of our methodology, which focused on exploring outcomes from lens of participants' experience, the community and societal transformations discussed in our findings echo recent theoretical dialogue about how music can provide space to reform and rehearse healthy relationships and be a community resource for ‘healthy publics.’^50^ ^(p1)^

The way this one community music initiative demonstrates positive health and well‐being outcomes across socioecological levels has important implications for health promotion more broadly. Embracing an expanded understanding of what might constitute a health promoting initiative can support health promotion practitioners and researchers to look beyond siloed interventions or campaigns that aim to address a single, ‘unhealthy’ behaviour, and explore holistic approaches that support personal development and supportive environments for thriving.^7^ Importantly, we need to move beyond mitigating consequences of health and social inequity and work towards addressing the social structures that cause this inequity.^6^ This study demonstrates how music may be uniquely placed to attend to some of these community and societal level drivers in small but significant ways. Building coalitions and collaborations across diverse sectors outside of health, including the arts and social sectors, is fruitful ground for health promotion researchers and practitioners seeking to find innovative ways to address health inequity.^49^, ^51^ When addressing the complex and wicked problems of our time, such as health inequity, health promotion can look to communities and empower existing work that is already making impact. Considering qualitative approaches to research and evaluation that centre community perspective and experiences will be an important part of this work.

Identifying and reinforcing community resources is a key starting point for good health promotion practice.^49^, ^52^ It is now well understood that addressing health inequity requires working *with* communities in strengths‐based ways to bolster capacity for individuals and communities to both define and achieve health and well‐being.^6^, ^53^ Our study demonstrates the importance of carefully considering community strengths and resources. Exploring how to bolster and build on these may be more effective, economical and sustainable than piloting new programs designed outside the community.^54^, ^55^

### 4.1 Strengths and limitations

Qualitative and ethnographic methodologies are increasingly being recognised as important in health research, including in health promotion evaluation.^56^, ^57^ Our methodology aimed to explore outcomes from the lens of participant experience and our methods identified outcomes across multiple levels in a complex social context; this could not have been achieved without long‐term engagement and relationship development with the community.^56^ Substantiating possible societal transformations discussed by participants was outside of the scope of this study, and not the aim of the article. Our methods allowed for contextual and in‐depth data that captured the perspectives and experiences of participants, illuminating how community music might work to support individual and collective well‐being. It will be important for future research in this area to explore in greater depth the community and societal level outcomes that community music initiatives might facilitate.

## 5 CONCLUSION

The study investigates an established community music initiative with positive health and well‐being outcomes across individual, community and societal levels. In doing so, this article suggests community music may be one useful approach for health promotion practice that seeks to work with communities in strengths‐based ways to address health inequity. Our findings highlight the potential for working collaboratively across sectors and the importance of harnessing a community's existing creative strengthens and resources for health equity work.

## FUNDING INFORMATION

This research was made possible through an Australian Research Council Future Fellowship (Creative Change Project, Professor Brydie‐Leigh Bartleet).

## CONFLICT OF INTEREST STATEMENT

The authors have no relevant financial or non‐financial interests to disclose.

## ETHICS STATEMENT

This study was approved by Griffith University's Human Research Ethics Committee (2020/679).

## Data Availability

Data sharing is not applicable to this article as no new data were created or analyzed in this study.
